# A transfer learning model with multi-source domains for biomedical event trigger extraction

**DOI:** 10.1186/s12864-020-07315-1

**Published:** 2021-01-07

**Authors:** Yifei Chen

**Affiliations:** School of Information Engineering, Nanjing Audit University, 86 West Yushan Road, Nanjing, China

**Keywords:** Event trigger recognition, Transfer learning, Adversarial networks, Multi-source domains

## Abstract

**Background:**

Automatic extraction of biomedical events from literature, that allows for faster update of the latest discoveries automatically, is a heated research topic now. Trigger word recognition is a critical step in the process of event extraction. Its performance directly influences the results of the event extraction. In general, machine learning-based trigger recognition approaches such as neural networks must to be trained on a dataset with plentiful annotations to achieve high performances. However, the problem of the datasets in wide coverage event domains is that their annotations are insufficient and imbalance. One of the methods widely used to deal with this problem is transfer learning. In this work, we aim to extend the transfer learning to utilize multiple source domains. Multiple source domain datasets can be jointly trained to help achieve a higher recognition performance on a target domain with wide coverage events.

**Results:**

Based on the study of previous work, we propose an improved multi-source domain neural network transfer learning architecture and a training approach for biomedical trigger detection task, which can share knowledge between the multi-source and target domains more comprehensively. We extend the ability of traditional adversarial networks to extract common features between source and target domains, when there is more than one dataset in the source domains. Multiple feature extraction channels to simultaneously capture global and local common features are designed. Moreover, under the constraint of an extra classifier, the multiple local common feature sub-channels can extract and transfer more diverse common features from the related multi-source domains effectively. In the experiments, MLEE corpus is used to train and test the proposed model to recognize the wide coverage triggers as a target dataset. Other four corpora with the varying degrees of relevance with MLEE from different domains are used as source datasets, respectively. Our proposed approach achieves recognition improvement compared with traditional adversarial networks. Moreover, its performance is competitive compared with the results of other leading systems on the same MLEE corpus.

**Conclusions:**

The proposed Multi-Source Transfer Learning-based Trigger Recognizer (MSTLTR) can further improve the performance compared with the traditional method, when the source domains are more than one. The most essential improvement is that our approach represents common features in two aspects: the global common features and the local common features. Hence, these more sharable features improve the performance and generalization of the model on the target domain effectively.

## Background

Recently, with the biomedical research development, an explosive amount of literature has been published online. As a result, it has brought a big challenge to the tasks of biomedical Text Mining (TM) for automatic identification and tracking of the new discoveries and theories in these biomedical papers [1–3]. Recognizing biomedical events in text is one of critical tasks, which refers to automatically extracting structured representations of biomedical relations, functions and processes from text [3]. Since the BioNLP’09 [4] and BioNLP’11 [5] Shared Tasks, event extraction has become a research focus, and many biomedical event corpora have sprung up, especially on molecular-level. For instance, a corpus from the Shared Task (ST) of BioNLP’09 [4] contains 9 types of frequently used biomolecular events. A corpus from the Epigenetics and Post-translational Modifications (EPI) task of BioNLP’11 [5] contains 14 protein entity modification event types and their catalysis. And another corpus consists of events relevant to DNA methylation and demethylation and their regulations [6]. Moreover, in order to obtain a more comprehensive understanding of biological systems, the scope of event extraction must be broadened from molecular-level reactions to cellular-, tissue- and organ-level effects, and to organism-level outcomes [7]. Hence, in MLEE corpus [8] wide coverage of events from the molecular level to the whole organism have been annotated with 19 event categories.

The structure of each event is defined through event triggers and their arguments. Hence, the most popular methods of event extraction contain two main steps: identifying the event triggers and then the arguments sequentially [9]. The first step, event trigger recognition, recognizing those verbal forms that indicate the appearances of events, is crucial to event extraction. Event extraction performance depends entirely on the recognized triggers. Previous study of Bj$\ddot {o}$rne et al. [10] clearly reveals that more than 20 points performance degradation is caused by the errors introduced by the use of predicted triggers rather than the gold standard triggers. A large number of methods have been proposed to predict the types of trigger words. Each word in an input sentence is assigned an event category label, or a negative label if it does not represent any event. Many machine learning-based methods, especially Artificial Neural Network (ANN) or deep learning-based methods, have been successfully applied to recognize event trigger words [11–13]. These methods mainly focus on improving the network construction to acquire various effective feature presentations from the text. The stronger feature learning capabilities of deep learning models improve trigger word recognition performance.

However, these deep learning-based approaches rely on large quantity and high quality annotated training data. Acquiring manually labeled data is both time consuming and expensive. It is not trivial to keep up to date with the annotations of expanding event types across wide coverage in biomedical literature, including molecular-, cellular-, tissue-, organ-, and organism-levels. As we have mentioned above, MLEE is one of this kind of corpus, which has 19 event categories. Among them, there are nearly 1000 annotations in the most annotated category, while there are less than 10 annotations in the least annotated category. Moreover, there are eight categories whose annotations are less than 100. Hence, the main issues of the dataset are lacking of labeled data and data imbalance, which will greatly degrade recognition performance. It is desirable to adopt other new techniques to learn a higher accuracy trigger recognizer with limited annotated and highly imbalanced training data. Recently, transfer learning (TL) has been proposed to tackle the issues [14], which has been successfully applied to many real world applications, including text mining [15, 16]. Briefly, the purpose of transfer learning is to achieve a task on a target dataset using some knowledge learned from a source dataset [14, 17]. These transfer learning methods mainly focus on obtaining more data from related source domains to improve the recognition performance. Through making use of transfer learning, the amount of data on the target dataset that needs manual annotation is reduced. Moreover, the generalization of the model on the target dataset can be improved. With transfer learning, a large amount of annotated data from related domains (such as the corpus of biomolecular event annotations, the corpus of Epigenetics and Post-translational Modifications (EPI) task, the corpus of DNA methylation and demethylation event annotations, and so on) is helpful to alleviate the shortage and imbalance problem of training data in the target task domain (such as the MLEE corpus).

Many methods of transfer learning have obtained remarkable results in many data mining and machine learning fields through transferring knowledge from source to target domains [18–20]. Among these transfer learning methods, adversarial training achieves great success recently [21], and attracts more and more researcher attention. Zhang et al. ([22]) introduces an adversarial method for transfer learning between two (source and target) Natural Language Processing (NLP) tasks over the same domain. A shared classifier is trained on the source documents and labels, and applied to target encoded documents. The proposed transfer method through adversarial training ensures that encoded features are task-invariant. Gui et al. ([23]) proposes a novel recurrent neural network, Target Preserved Adversarial Neural Network (TPANN) to do Part-Of-Speech (POS) tagging. The model can learn the common features between source (out-of-domain labeled data) domain and target (unlabeled in-domain data, and labeled in-domain data) domain, simultaneously preserve target domain-specific features. Chen et al. ([24]) proposes an Adversarial Deep Averaging Network (ADAN) for cross-Lingual sentiment classification. ADAN has a sentiment classifier and an adversarial language discriminator to take input from a shared feature extractor to learn hidden representations. ADAN transfers the knowledge learned from labeled data on a resource-rich source language to low-resource languages where only unlabeled data exist. Kim et al. ([25]) proposes a cross-lingual POS tagging model that utilizes common features to enable knowledge transfer from other languages, and private features for language-specific representations.

Traditional transfer learning models were designed to transfer knowledge from a single source domain to the target domain. In the practical application of biomedical trigger recognition, we can access to datasets from multiple domains. This is also the case in many other applications. Hence, some multi-source domain transfer learning approaches are proposed. Chen and Cardie ([26]) proposes a Multinomial Adversarial Network (MAN) for multi-domain text classification. MAN learns features that are invariant across multiple domains. The method extracts sharable features between source domains and the target domain globally. Some multi-task learning methods with multiple source domains are involved. Chen et al. ([27]) proposes adversarial multi-criteria learning for Chinese word segmentation by integrating shared knowledge from multiple segmentation criteria. The approach utilizes adversarial strategy to make sure the shared layer can extract the common underlying and criteria-invariant features, which are suitable for all the criteria. Liu et al. ([28]) proposes an adversarial multi-task learning framework for text classification, in which the feature space is divided into the shared and private latent feature space through adversarial training. These methods are dedicated to extract shared features between source domains and the target domains globally, which are invariant among all the available domains. They don’t concern the distinct importance of each source to the target domain. On the other hand, Guo et al. ([29]) puts forward an approach only from the aspect of capturing the relation between the target domain and each source domain to extract common features.

Generally, these models separate the feature space into the shared and private space. The features from the private space are used to store domain-dependent information, while the ones from the shared space are extracted to capture domain-invariant information that is transferred from the source domain. We can assume that if there are multiple datasets from different but related source domains available, it may bring more transferred knowledge and produce more performance improvement. The major limitation of these methods is the fact that they cannot be easily extended to make full use of datasets from multiple source domains. With the division methods, the feature space that can be globally shared with the target domain and all the source domains may be limited. These globally shared features are invariant to all these domains. It is no guarantee that there are more sharable features do not exist outside these global shared features. Hence, some useful sharable features could be ignored. Our idea is that a suitable shared feature space should contain more common information besides the global shared features. To address the problem, we propose a method to compensate for the deficiency. In our method, common (shared) features are composed of two parts: the global common (shared) features and the local common (shared) features. The global common features are extracted and domain-invarian among all the source domains and the target domain, while the local common features are extracted between a pair of single source domain and the target domain. We attempt to combine the capabilities of sharable features extracted from different aspects simultaneously. To achieve this goal, we adopt adversarial networks into a multi-channel feature extraction framework to transfer knowledge from multiple source domains more comprehensively. This provides us with more feature information from relevant datasets.

Our aim in this study is to transfer the trigger recognition knowledge from multiple source domains to the target domain more comprehensively. In summary, the contributions of this paper are as follows:

- We propose a improved Multi-Source Transfer Learning-based Trigger Recognizer (MSTLTR) framework to incorporate data from multiple source domains by using adversarial network-based transfer learning. To our knowledge, no reported research has applied multi-source transfer learning to make the best use of related annotated datasets to find the sharable information in biomedical trigger word recognition task. The MSTLTR framework can adapt to the situation of zero to multiple source domain datasets.
- We design multiple feature extraction channels in MSTLTR, which aim to capture global common features and local common features simultaneously. Moreover, under the constraint of an extra classifier, the multiple local common feature sub-channels can extract and transfer more diverse common features from the related multi-source domains effectively. Finally, through feature fusion, the influence of important features will be magnified, on the contrary, the impact of unimportant features will be reduced.
- Comprehensive experiments on the event trigger recognition task confirm the effectiveness of the proposed MSTLTR framework. Experiments show that our approach improves the recognition performance over the traditional division models further. Moreover, its performance is competitive compared with the results of other leading systems on the same corpus.

The rest of this paper is organized as follows. A detailed description of the proposed improved Multi-Source Transfer Learning-based Trigger Recognizer (MSTLTR) framework is introduced in “Methods” section. “Results” section describes the used biomedical corpora and experimental settings, and all the experimental results. Then “Discussion” section presents in-depth analysis. Finally, we present a conclusion and future work in “Conclusions” section.

## Results

### Corpus description

An in-depth investigation is carried out to compare the performance of our proposed Multi-Source Transfer Learning-based Trigger Recognizer, MSTLTR. The dataset *Data*_*MLEE*_ is used as the target domain dataset. With varying degrees of label overlapping, *Data*_*ST*09_,*Data*_*EPI*_,*Data*_*ID*_ and *Data*_*DNAm*_ are used as the source domain datasets.

#### *Data*_*MLEE*_

The MLEE corpus [8] is used to train and test our MSTLTR model as a target dataset. The corpus is taken from 262 PubMed abstracts focusing on tissue-level and organ-level processes, which are highly related to certain organism-level pathologies. In *Data*_*MLEE*_, 19 event types are chosen from the GENIA ontology, which can be classified into four groups: anatomical, molecular, general and planned. Our task is to identify the correct trigger type of each word. Hence, there are 20 tags in the target label set, including a negative one. The named entity and trigger types annotated in the corpus are illustrated in Table 1. In the trigger types of *Data*_*MLEE*_, ten labels overlapped with source datasets are marked using ‘*’. Moreover, the corresponding number of triggers of the overlapped types in both *Data*_*MLEE*_ and each source corpus, and also the proportions of these numbers per total number of triggers in each corpus are shown in Table 2. In the target domain dataset *Data*_*MLEE*_, the overlapped trigger with the highest proportion is “Positive regulation”, and its proportion is ‘966/5407’, i.e. 18%. On the other hand, the overlapped trigger with the lowest proportion is “Dephosphorylation”, and its proportion is only ‘3/5407’, i.e. 0.06%. There is a big gap between them. At the same time, we can see that the trigger “Phosphorylation” from the target dataset overlaps in all the source domain datasets. “Dephosphorylation” overlaps only in one source domain dataset *Data*_*EPI*_. And the remaining triggers only overlap in the two source domain datasets, *Data*_*ST*09_ and *Data*_*ID*_. All the statistics of sentences, words, entities, triggers and events in the training, development and test sets are presented in Table 3.

**Table 1:** Named entity and trigger types in *Data*_*MLEE*_, the target domain dataset. In the trigger types of *Data*_*MLEE*_, the labels overlapped with source domain datasets are marked using ‘*’

**Corpus**	**Named entity type**	**Trigger type**
*Data*_*MLEE*_	Gene or gene product	Cell proliferation, Planned process
	Drug or compound	Development, Synthesis
	Developing anatomical structure	Blood vessel develop
	Organ, Tissue	Growth, Death
	Immaterial anatomical entity	Breakdown, Remodeling
	Anatomical system	Regulation*, Localization*
	Organism, Cell	Binding*, Gene expression*
	Pathological formation	Transcription*
	Organism subdivision	Protein catabolism*
	Multi-tissue structure	Phosphorylation*
	Cellular component	Dephosphorylation*
	Organism substance	Positive regulation*
		Negative regulation*

**Table 2:** The detailed statistics of triggers of overlapped types between each source corpus and the target corpus, including (1) the numbers of triggers of overlapped types between each source corpus and the target corpus, (2) and the proportions of these numbers per total number of triggers in each corpus

**Overlapped trigger type**	**Target**	**Source**	**Source**	**Source**	**Source**
	***Data***_***MLEE***_	***Data***_***ST*09**_	***Data***_***EPI***_	***Data***_***DNAm***_	***Data***_***ID***_
Regulation	540/5407	1026/10270	-	-	187/2155
Localization	415/5407	268/10270	-	-	43/2155
Binding	158/5407	1007/10270	-	-	125/2155
Gene expression	342/5407	2374/10270	-	-	347/2155
Transcription	23/5407	654/10270	-	-	47/2155
Protein catabolism	24/5407	120/10270	-	-	27/2155
Phosphorylation	29/5407	231/10270	112/2038	3/707	54/2155
Dephosphorylation	3/5407	-	3/2038	-	-
Positive regulation	966/5407	2379/10270	-	-	298/2155
Negative regulation	683/5407	1311/10270	-	-	180/2155

**Table 3:** Statistics of sentences, words, entities, triggers and events in the dataset *Data*_*MLEE*_, including the training set, the development set, and the test set, respectively

**Item**	**Training**	**Development**	**Test**
Sentences	1271	457	880
Words	27,875	9,610	19,103
Entities	4147	1431	2713
Triggers	2685	913	1809
Events	3,296	1,175	2260

#### *Data*_*ST*09_

This corpus is taken from the Shared Task (ST) of BioNLP challenge 2009 [4] and contains training and development sets, including 950 abstracts from PubMed. It is used to train our MSTLTR as a source dataset. In this corpus, 9 event types are chosen from the GENIA ontology involving molecular-level entities and processes, which can be categorized into 3 different groups: simple events, binding events and regulation events. The named entity and trigger types annotated in the corpus are illustrated in Table 4. In the trigger types of *Data*_*ST*09_, the labels overlapped with the target dataset are marked using ‘*’. We can see that it is nested in the label set of the target domain with 9 overlapped labels. The training and development sets are combined as a source domain dataset *Data*_*ST*09_. Moreover, the corresponding number of triggers of the overlapped types in both *Data*_*ST*09_ and the target corpus, and also the proportions of these numbers per total number of triggers in each corpus are shown in Table 2. In the source domain dataset *Data*_*ST*09_, the overlapped trigger with the highest proportion is “Positive regulation”, and its proportion is ‘2379/10270’, i.e. 23%. On the other hand, the overlapped trigger with the lowest proportion is “Protein catabolism”, and its proportion is only ‘120/10270’, i.e. 1%. All the statistics of sentences, words, entities, triggers and events in *Data*_*ST*09_ are shown in Table 5.

**Table 4:** Named entity and trigger types in *Data*_*ST*09_. In the trigger types of *Data*_*ST*09_, the labels overlapped with *Data*_*MLEE*_ are marked using ‘*’

**Corpus**	**Named entity type**	**Trigger type**
*Data*_*ST*09_	Protein	Gene expression*
		Transcription*, Binding*
		Protein catabolism*
		Phosphorylation*
		Localization*, Regulation*
		Positive regulation*
		Negative regulation*

**Table 5:** Statistics of sentences, words, entities, triggers and events in the source domain datasets, *Data*_*ST*09_,*Data*_*EPI*_,*Data*_*ID*_ and *Data*_*DNAm*_, respectively

**Source dataset**	**Sentences**	**Words**	**Entities**	**Triggers**	**Events**
*Data*_*ST*09_	10,761	269,861	16,315	10270	13,560
*Data*_*EPI*_	7,827	170,809	10,094	2038	2,453
*Data*_*DNAm*_	1,305	32,510	1,964	707	1,034
*Data*_*ID*_	3,412	83,063	8,501	2155	2,779

#### *Data*_*EPI*_

This corpus is taken from the Epigenetics and Post-translational Modifications (EPI) task of BioNLP challenge 2011 [5] and contains training and development sets, including 800 abstracts relating primarily to protein modifications drawn from PubMed. It is also used to train our MSTLTR as a source domain dataset. In this corpus, there are 15 event types, including 14 protein entity modification event types and their catalysis. The named entity and trigger types annotated in the corpus are illustrated in Table 6. In the trigger types of *Data*_*EPI*_, the labels overlapped with the target dataset are marked using ‘*’. There are only 2 labels are overlapped, which is weakly related with the target domain. The training and development sets are combined as a source domain dataset *Data*_*EPI*_. Moreover, the corresponding number of triggers of the overlapped types in both *Data*_*EPI*_ and the target corpus, and also the proportions of these numbers per total number of triggers in each corpus are shown in Table 2. In the source domain dataset *Data*_*EPI*_, one overlapped trigger is “Phosphorylation”, and its proportion is ‘112/2038’, i.e. 5%. The other overlapped trigger is “Dephosphorylation”, and its proportion is only ‘3/2038’, i.e. 0.1%. All the statistics of sentences, words, entities, triggers and events in *Data*_*EPI*_ are shown in Table 5. The number of annotated triggers in *Data*_*EPI*_ is less than that in the *Data*_*ST*09_, annotating the more event types.

**Table 6:** Named entity and trigger types in *Data*_*EPI*_. In the trigger types of *Data*_*EPI*_, the labels overlapped with *Data*_*MLEE*_ are marked using ‘*’

**Corpus**	**Named entity type**	**Trigger type**
*Data*_*EPI*11_	Protein	Hydroxylation, Dehydroxylation
		Phosphorylation*, Deglycosylation
		Dephosphorylation*, Catalysis
		Ubiquitination, Acetylation
		Deubiquitination
		DNA methylation
		DNA demethylation
		Glycosylation, Deacetylation
		Methylation, Demethylation

#### *Data*_*DNAm*_

This corpus consists of abstracts relevant to DNA methylation and demethylation events and their regulation. The representation applied in the BioNLP ST on event extraction was adapted [6]. It is also used to train our MSTLTR as a source dataset. The named entity and trigger types annotated in the corpus are illustrated in Table 7. In the trigger types of *Data*_*DNAm*_, the only one label overlapped with the target dataset are marked using ‘*’. The training and development sets are combined as a source domain dataset *Data*_*DNAm*_. From Table 2, in the source domain dataset *Data*_*DNAm*_, the only overlapped trigger is “Phosphorylation”, and its proportion is ‘3/707’, i.e. 0.4%. All the statistics of sentences, words, entities, triggers and events in *Data*_*DNAm*_ are shown in Table 5.

**Table 7:** Named entity and trigger types in *Data*_*DNAm*_. In the trigger types of *Data*_*DNAm*_, the labels overlapped with *Data*_*MLEE*_ are marked using ‘*’

**Corpus**	**Named entity type**	**Trigger type**
*Data*_*DNAm*_	Protein	DNA methylation
		DNA demethylation
		Phosphorylation*
		Ubiquitination
		Methylation
		Deacetylation

#### *Data*_*ID*_

This corpus is taken from the Infectious Diseases (ID) task of BioNLP challenge 2011 [5], drawn from the primary text content of recent 30 full-text PMC open access documents focusing on the biomolecular mechanisms of infectious diseases. It is also used to train our MSTLTR as a source dataset. In this corpus, 10 protein entity modification event types are chosen. The core named entity and trigger types annotated in the corpus are illustrated in Table 8. In the trigger types of *Data*_*ID*_, the labels overlapped with the target dataset are marked using ‘*’. Same as *Data*_*ST*09_, there are 9 overlapped trigger labels. The difference is that *Data*_*ID*_ has one label “Process” that does not belong to the target domain. The training and development sets are combined as a source domain dataset *Data*_*ID*_. From Table 2, in the source domain dataset *Data*_*ID*_, the overlapped trigger with the highest proportion is “Gene expression”, and its proportion is ‘347/2155’, i.e. 16%. On the other hand, the overlapped trigger with the lowest proportion is “Protein catabolism”, and its proportion is only ‘27/2155’, i.e. 1%. All the statistics of sentences, words, entities, triggers and events in *Data*_*ID*_ are shown in Table 5. In addition to “protein”, the *Data*_*ID*_ defines four more types of core entities, including “two-component-system”, “regulon-operon”, “chemical” and “organism”.

**Table 8:** Named entity and trigger types in *Data*_*ID*_. In the trigger types of *Data*_*ID*_, the labels overlapped with *Data*_*MLEE*_ are marked using ‘*’

**Corpus**	**Named entity type**	**Trigger type**
*Data*_*ID*_	Protein	Gene expression*
	two-component-system	Transcription*
	regulon-operon	Protein catabolism*
	chemical	Phosphorylation*
	organism	Localization*
		Binding*
		Process
		Regulation*
		Positive regulation*
		Negative regulation*

### Implementation details

All of the experiments are implemented using the Tensorflow library [30]. Batch size is 20 for all the tasks from no matter what domain the recognition task comes from. We tune the pre-trained word embedding vector *E*^*w*^ to 200 dimensions, character embedding vector *E*^*c*^ to 100, POS embedding vector *E*^*p*^ to 50, named entity type embedding vector *E*^*e*^ to 10, and dependency tree-based word embedding vector *E*^*d*^ to 300 dimensions for all the source domains and the target domain. BiLSTMs are used in the private, global common and local common feature extraction components. In particular, they are all with a hidden state dimension of 300 (150 for each direction). In the feature fusion layer, the fully-connected units are 600. Hyper-parameters are tuned using training and development sets through cross-validation and then the final model is trained on the combined set of the optimal ones. The trade-off hyper-parameters are set to *α*_1_=0.04,*α*_1_=0.01, and *β*=0.1. In order to avoid overfitting, dropout with a probability 0.5 is applied in all components.

### Performance assessment

We measure the performance of the trigger recognition system in terms of the *F*1-measure. The *F*1 is determined by a combination of precision and recall. Precision is the ratio of the real positive instances to the positive instances in the classification results of the model. Recall is the ratio of the real positive instances in the classification results of the model to the real positive instances in the data. They are defined as follows:

1 $$ F1-measure = \frac{2Precision \times Recall}{Precision+Recall}  $$

2 $$ Precision = \frac{TP}{TP+FP}  $$

3 $$ Recall = \frac{TP}{TP+FN}  $$

where *TP* is the number of the instances that are correctly classified to a category, *FP* is the number of the instances that are misclassified to a category, and *FN* is the number of the instances misclassified to other categories.

### Transfer learning performance

In this section, comprehensive experiments is carried out to study the performance of our proposed Multi-Source Transfer Learning-based Trigger Recognizer, MSTLTR. First of all, we will analyze the impact of different combinations of source domain datasets on our transfer learning-based model through a group of experiments. Then, based on these experiments, the performance of the best model is compared with other leading systems.

The first group of experiments is used to compare the performance changes of our transfer learning model under different number of source domain datasets. For convenience, all source datasets are numbered from *S*1 to *S*4 in the order of *Data*_*ST*09_,*Data*_*EPI*11_,*Data*_*DNAm*_ and *Data*_*DI*_. The results are summarized in Table 9, which can be divided into 4 modes, including “No source”, “One source”, “Two sources” and “Multi-source”. In the first “No Source” mode, the trigger recognition result without transfer learning is displayed, which is a Basic Model. The more detailed description of the Basic Model is in “Basic model” section. Then in the second “One Source” mode, all the transfer learning model results using only one source dataset are listed. The third mode, “Two Sources”, illustrates the results under all the combination of 2 source datasets. However, there are many combinations. Considering the limited space, we only list the combinations of the best single source dataset (*S*1) and other datasets. Finally, “Multi-Source” mode shows the results of 3 and 4 source datasets. The illustrated 3 source dataset results are obtained based on the best “Two Sources” results. In each mode, the average results of all possible combinations of the source domains are listed by “AVG”.

**Table 9:** Detailed results achieved by the proposed MSTLTR Model with different number of source datasets. All source datasets are numbered from *S*1 to *S*4 in the order of *Data*_*ST*09_,*Data*_*EPI*11_,*Data*_*DNAm*_ and *Data*_*DI*_. In the first “No Source” mode, the trigger recognition result without transfer learning is displayed. Then in the second “One source” mode, all the results using only one source dataset are listed. In the third “Two sources” mode, only the results of the combinations of the best single source dataset (*S*1) and other datasets are listed. Finally, “Multi-source” mode shows the results of multiple source domain transfer learning, including 3 and 4 source datasets. The illustrated 3 source dataset results are obtained based on the best “Two Sources” results. In each mode, the average results of all possible combinations of the source domains are listed by “AVG”

**Mode**	**Source domain**	**Precision**	**Recall**	**F1-measure**
No Source	- (Basic Model)	79.47	77.23	78.34
One Source	*Data*_*ST*09_(*S*1)	82.25	77.89	80.01
	*Data*_*EPI*11_(*S*2)	81.74	76.60	79.09
	*Data*_*DNAm*_(*S*3)	81.99	76.44	79.12
	*Data*_*DI*_(*S*4)	82.80	77.24	79.92
	AVG	-	-	79.53
Two Sources	*S*1+*S*2	81.79	78.78	80.26
	*S*1+*S*3	83.28	77.77	80.43
	*S*1+*S*4	83.16	78.56	80.80
	AVG	-	-	80.24
Multi-Source	*S*1+*S*2+*S*4	83.62	78.36	80.90
	*S*1+*S*3+*S*4	84.10	78.36	81.13
	*S*1+*S*2+*S*3+*S*4(MSTLTR Model)	83.96	79.89	81.88
	AVG	-	-	81.25

From the results we can see that no matter how many source datasets are utilized, our proposed MSTLTR can improve the trigger recognition performance. Further, the more source datasets are used, the more performance improvements can be achieved. Compared with the “No Source” result, which is achieved without using transfer learning, “One Source” can increase the performance by 1.19% on average, “Two Sources” can increase the performance by 1.9% on average, and “Multi-Source” can increase the performance by 2.91% on average. In the best case, when 4 source domain datasets are used, the performance improvement can reach 3.54%. This improvement is due to the fact that with multiple source domain datasets, more features are transferred to the target domain, signifying more effective knowledge sharing. It is worth noting there are improvements in both precision and recall, which refer to the ability of MSTLTR to identify more positive triggers. Higher precision and recall signify identification of more potential biomedical events during the subsequent processing phase, which is important for the ultimate event extraction application.

If we make a more detailed analysis, it is shown that the amount of knowledge that can be transferred from the source datasets is different, when they have different degrees of overlap with the target dataset. In the “One Source” mode, the source datasets *Data*_*ST*09_ and *Data*_*DI*_ having 9 overlapping event triggers with the target dataset can both improve the performance more than the source datasets *Data*_*EPI*11_ and *Data*_*DNAm*_ having just 2 and 1 overlapping event triggers, respectively. The more related the source dataset is to the target dataset, the more effective the transfer learning is. However here, the difference between them is not significant.

### MSTLTR compared with other trigger recognition systems

Then, based on the best setting of the previous group of experiments, we compare the performance of the proposed Multi-Source Transfer Learning-based Trigger Recognizer, MSTLTR, with other leading systems on the same *Data*_*MLEE*_ dataset. The detailed *F*1-measure results are illustrated in Table 10.

**Table 10:** Detailed performance results achieved by the proposed MSTLTR and the other leading trigger recognition systems

**Trigger recognition system**	**Precision**	**Recall**	**F1-measure**
SVM feature-based System [8]	81.44	69.48	75.67
SVM-based System [31]	80.60	81.60	78.32
Neural Network-based System [32]	71.04	84.60	77.23
CNN-based Neural Network System [33]	80.67	76.76	78.67
RNN-based Neural Network System [34]	79.78	78.45	79.11
Attention-based Neural Network System [35]	81.33	79.48	80.39
Tree-base Neural Network System [11]	81.12	79.15	80.28
Convolutional Highway Neural Network System [12]	80.06	81.25	80.57
Hybrid Neural Network System [13]	80.03	81.54	80.66
Joint-GATE-Sentence Neural Network System [36]	81.58	81.08	81.33
Joint-GATE-Document Neural Network System [36]	82.11	82.53	82.32
BioBERT-based Neural Network System [37]	79.48	83.76	81.57
Our MSTLTR System	83.96	79.89	81.88

Pyysalo et al. [8] defines an SVM feature-based System with rich hand-crafted features to recognize triggers in the text. Zhou et al. [31] also defines an SVM-based System with word embeddings and hand-crafted features. Nie et al. [32] proposes a word embedding-assisted Neural Network-based System to model semantic and syntactic information in event trigger identification. Wang et al. [33] defines a window-based convolution neural network classifier, a CNN-based Neural Network System. Rahul et al. [34] proposes a method that uses a recurrent neural network (RNN-based Neural Network System) to extract higher-level sentence features in trigger identification. Li et al. [35] proposes a novel contextual label sensitive gated network for biomedical event trigger extraction, as well as attention mechanism (Attention-based Neural Network System) to get more focused representations of dependency-based semantic information. Hao et al. [11] proposes a recursive neural network to represent the whole dependency tree globally (Tree-base Neural Network System), to better incorporate dependency-based syntax information. Chen et al. [12] presents an end-to-end Convolutional Highway Neural Network System and extreme learning machine (CHNN-ELM) framework to detect biomedical event triggers. Diao et al. [13] proposes a Hybrid Neural Network System which consists of Fine grained Bidirectional Long Short Term Memory (FBi-LSTM) and Support Vector Machine (SVM) to deal with the event trigger identification. Zhang et al. [36] proposes a variational neural approach, which can take advantage of latent topics underlying documents for biomedical event extraction. Joint-GATE-Document model is the proposed model that jointly trains document-level latent topics, obtained through a designed document-level neural topic model (NTM), and trigger detection. Joint-GATE-Sentence is a similar model, but latent topics are learned on the sentence-level. Lee et al. [37] proposes a domain-specific language representation model BioBERT, which is pre-trained on large-scale biomedical corpora. It uses bidirectional encoder representations from transformers, which has almost the same architecture of BERT [38]. BioBERT-base Neural Network System is built using the source code 1 on *Data*_*MLEE*_ through fine-tuning based on the pre-trained weights BioBERT-Base v1.1. The optimal combination of hyper-parameters and pre-trained weights is obtained using cross-validation.

From the results in Table 10, we can draw following conclusions:

- The neural network methods outperform the feature-based methods on the average. In Table 10, only the first SVM feature-based System uses purely hand-crafted features. The second SVM-based System uses word embeddings learned by neural language modeling in addition to the rich hand-crafted features. The remaining neural network-based systems use distributional representations of words, rather than manual features. All these neural network methods have brought different degrees of performance improvement. F1-measure can be increased by 6.65 at most and 1.56 at least.
- Through careful structure design, recognition performance of neural network system is better. In the neural network systems, compared with the basic CNN or RNN network structures (Neural Network-based System, CNN-based Neural Network System and RNN-based Neural Network System), careful designs of tree structure (Tree-base Neural Network System), attention mechanism (Attention-based Neural Network System), convolutional Highway structure (Convolutional Highway Neural Network System), hybrid structure (Hybrid Neural Network System), document-level neural topic structure (Joint-GATE-Document Neural Network System), transfer learning from transformers (BioBERT-based Neural Network System) and transfer learning from adversarial networks (Our MSTLTR System) have brought the performance enhancement. F1-measure can be increased by 5.09 at most and 1.17 at least.
- Compared with Joint-GATE-Document Neural Network System, the F1-measure performance of our MSTLTR System is 0.44 lower, but it is still a competitive result with a higher precision. In Joint-GATE-Document Neural Network System, one of the important factors for performance improvement is the designed document level neural topic model, which extracts document-level context information. Compared with that, Joint-GATE-Sentence Neural Network System model mentioned in [36], using sentence-level context information, has a F1-measure 0.55 lower than our MSTLTR System. Another important factor is the use of BERT word embeddings. In addition to the word2vec model we all use, they also employ BERT embeddings. According to their comparison, “without the BERT embeddings, it leads to mostly 1.55 degradation” of the model performance.
- Similar to BioBERT-based Neural Network System, we all use the idea of transfer learning. And our models both are pre-trained on the source domain datasets, and work on the target domain through fine-tuning. However, we use different mechanisms and network structures to realize transfer learning. BioBERT has almost the same architecture of BERT, which consists of 12 layers of multi-head self-attention networks. BioBERT is pre-trained on PubMed abstracts (PubMed) and PubMed Central full-text articles (PMC). Then, all the pre-trained network weights are used to represent features and be fine-tuned on the target domain. For our MSTLTR System, adversarial network is used to transfer knowledge. Our MSTLTR System is pre-trained on some relevant source domain data with overlapping labels. Then, only common features (including global common and local common features), not all the features, are fine-tuned on the target domain. The F1-measure of our MSTLTR System is 0.31 higher than that of BioBERT-based Neural Network System.
- Our MSTLTR System has the highest precision and competitive F1-measure values. However, the recall is lower than that of some systems. Then the recall needs to be further improved in our system, because that higher recall will bring more possible triggers available in the following step in the process of biomedical event extraction.

## Discussion

### Effect of common features

In this section, we take a closer look at the impact of different scope of feature channels on the trigger recognition performance. The features of MSTLTR model are provided by feature extraction channels, including a private feature channel, a global common feature channel and multiple local common feature sub-channels. All the trigger recognition models used to compare their results are illustrated in Table 11. First, “Model I” is the Basic Model without using any common features brought by transfer learning, which is a baseline performance here. Then, “Model II”s are the models just using global common features, which is a traditional adversarial method of transfer learning. The four models from “Model II-1” to “Model II-4” are built using 1 to 4 source datasets respectively. The combinations of source domain datasets adopt the modes that can achieve the best results in Table 9. Hence, “Model II-1” is built using source domain dataset *S*1, “Model II-2” is built using *S*1+*S*4, “Model II-3” is built using *S*1+*S*3+*S*4, and “Model II-4” is built using *S*1+*S*2+*S*3+*S*4 respectively. Finally, based on all the 4 source datasets, “Model III”, which is our proposed MSTLTR model, utilizes the global common and local common features simultaneously.

**Table 11:** Detailed results achieved by using different scope of feature channels of MSTLTR. The following situations are included: no shared features are used; only global shared features from 1 to 4 source domain datasets are used; both global common and local common features from 4 source domain datasets are used

**Models**	**Precision**	**Recall**	**F1-measure**
Model I: no common feature (Basic Model)	79.47	77.23	78.34
Model II-1: global common features (Single Source)	82.25	77.89	80.01
Model II-2: global common features (Two Sources)	82.44	79.10	80.74
Model II-3: global common features (Three Sources)	82.75	78.35	80.49
Model II-4: global common features (Four Sources)	82.40	77.71	79.99
Model III: Model II-4 + local common features (MSTLTR)	83.96	79.89	81.88

From the results in Table 11, we can get two main conclusions about global and local common features. First, no matter extracted from how many source datasets, the global common features can always improve the recognition performance. The global common features, extracted from a trained adversarial network, are domain-invariant, which can effectively improve the performance of recognition on the basis of private features. The global common features extracted using a single source domain increase *F*1-measure by 1.67. At the same time, from the results of the four “Model II”s we can see that, the model “Model II-4” trained through the most source datasets is not the best. On the contrary, the model “Model II-2” trained using two source datasets can obtain the best performance. In other words, more source data from different domain datasets may not bring more domain-invariant features. The global common feature channel is built to extract the domain-invariant features across all the target and source domain datasets. As the number of source domain datasets keeps increasing, the total domain-invariant features among them that can be extracted will decrease. In our system, some of common features will be provided and enhanced by local common feature channel. Therefore, when only global common features are used, the “Model II-2” obtains the best performance with the most global common features. The performance of “Model II-4” decreases with less global common features.

Second, based on global common features, adding the local common features can further improve recognition performance. Each local common feature sub-channel is also trained on an adversarial network, which can bring more transferred local common features between the target domain and certain source domain datasets. Moreover, an extra classifier is designed to prevent local common features of different source domains from interfering with each other. Hence, they can effectively provide different emphases from different domains to supplement the global common features. After feature fusion, the global common features are enhanced by the local common features. The influence of important features will be magnified, and the impact of unimportant features will be reduced. When “Model II-4” incorporates the local common features, the performance is further improved by 1.89.

### Quantity effect of target data

**Fig. 1:**
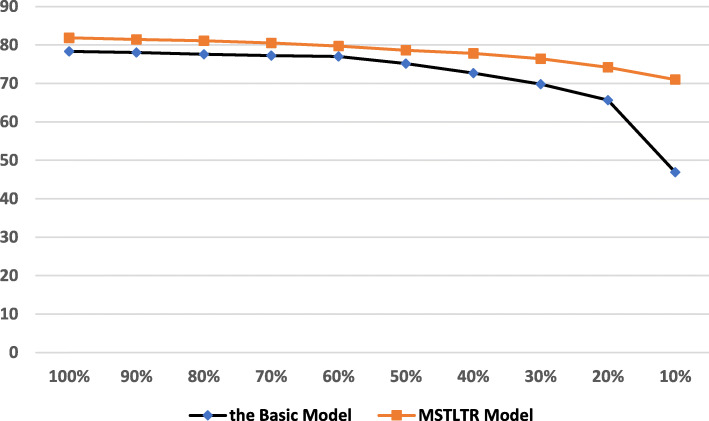
The quantity effect of the target domain data *Data*_*MLEE*_

It is important to analyze the effect of the quantity of the target domain data. We keep the size of the 4 source datasets unchanged, and gradually change the size of the target dataset. The changes in MSTLTR Model results are shown as broken line diagrams in Fig. 1, with the ratio as 100%, 90%, 80%, 70%, 60%, 50%, 40%, 30%, 20% and 10% of the original target dataset *Data*_*MLEE*_. In order to ensure that in each ratio, the proportion of instances among each category remains the same as the original target dataset *Data*_*MLEE*_, we adopt the stratified sampling method. Hence, the number of instances in each category including overlapped categories is reduced by the percentage of sampling. Then on each ratio of target dataset, we compare the *F*1-measure results with those of the Basic Model without transfer learning. We can find that under the condition of different quantities of target datasets, the performance results of MSTLTR Model are always better than those of the Basic Mode. As the amount of target data decreases, the recognition performance of the Basic Model initially decreases steadily. As the amount of target data continues to decrease, its performance drops significantly. From 100% to 10% of the original target data, the performance declines by 31.45. On the other hand, for MSTLTR Model, when the amount of target data is equally reduced, the decline of recognition performance becomes more gentle. The performance only declines by 10.88 from 100% to 10% of the original target data. Therefore, when the data in the target dataset is very rare, our model shows greater generalization ability.

### Error analysis

Finally, we will analyze the recognition performance of our MSTLTR Model on each category in more detail. From the metrics in Table 12 we can notice that compared with the Basic Model without using transfer learning, our MSTLTR Model has improved the performance of trigger recognition in 18 out of 19 categories. This includes categories such as “Remodeling”, “Synthesis”, “Transcription”, “Protein catabolism” and “Phosphorylation”. They’re the triggers that are short of labeling in the dataset, whose annotation sizes are from 10 to 50. A detailed list of types and sizes of trigger words of *Data*_*MLEE*_ is in Table 13. Among these triggers, “Transcription”, “Protein catabolism” and “Phosphorylation” overlap with the label sets of the source domain datasets, while “Remodeling” and “Synthesis” do not overlap with any label set at all. Therefore, to a certain extent, our model has the effective transfer ability to improve the recognition ability of rare trigger types “Remodeling” and “Synthesis”. However, the results of the trigger type “Dephosphorylation” are all zeroes regardless of the models, whose recognition performance has not been improved at all. The main reason is that there are only 3 “Dephosphorylation” instances in *Data*_*MLEE*_. Although “Dephosphorylation” is an overlapped type between the target and source domain datasets, it still lacks adequate training and test instances. Therefore, despite the use of transfer learning of MSTLTR Model, the recognition results of “Dephosphorylation” are still zeroes under the situation. This is a limitation of our transfer learning approach that it cannot transfer enough knowledge from source domains for labelling the very rare trigger types.

**Table 12:** Detailed results achieved by the proposed MSTLTR Model and the Basic Model on *Data*_*MLEE*_. The Basic Model is trained only on the training and development sets of *Data*_*MLEE*_ without transfer learning. MSTLTR Model is jointly trained on all available source domain datasets and the training and development sets of the target dataset *Data*_*MLEE*_ using proposed transfer learning approach. The two models are tested on the test set of *Data*_*MLEE*_

**Trigger type**	**Basic model**			**MSTLTR model**		
	**P**	**R**	**F1**	**P**	**R**	**F1**
Cell proliferation	83.33	81.40	82.35	85.37	81.40	83.33
Development	74.51	77.55	76.00	79.59	79.59	79.59
Blood vessel develop	98.64	93.87	96.20	99.66	93.87	96.68
Growth	88.89	85.71	87.27	91.23	92.86	92.04
Death	66.67	81.08	73.17	74.36	78.38	76.32
Breakdown	73.68	63.64	68.29	83.33	68.18	75.00
Remodeling	75.00	30.00	42.86	83.33	50.00	62.50
Synthesis	33.33	25.00	28.57	80.00	100.00	88.89
Gene expression	85.40	88.64	86.99	89.13	93.18	91.11
Transcription	50.00	16.67	25.00	100.00	50.00	66.67
Protein Catabolism	0.0	0.0	0.0	100.00	20.00	33.33
Phosphorylation	75.00	100.00	85.71	100.00	100.00	100.00
Dephosphorylation	0.0	0.0	0.0	0.0	0.0	0.0
Localization	76.81	79.70	78.23	86.61	82.71	84.62
Binding	82.69	75.44	78.90	83.64	80.70	82.14
Regulation	65.13	61.35	63.18	66.84	63.29	65.01
Positive regulation	80.56	82.86	81.69	82.91	83.17	83.04
Negative regulation	76.67	75.10	75.88	83.91	78.78	81.26
Planned process	66.67	57.14	61.54	73.78	61.73	67.22
TOTAL	79.47	77.23	78.34	83.96	79.89	81.88

**Table 13:** List of types and sizes of trigger words in *Data*_*MLEE*_

	**Trigger type**	**Size in*Data***_***MLEE***_
Anatomical	Cell proliferation	125
	Development	300
	Blood vessel develop	845
	Growth	163
	Death	93
	Breakdown	67
	Remodeling	32
Molecular	Synthesis	17
	Gene expression	342
	Transcription	24
	Protein catabolism	23
	Phosphorylation	29
	Dephosphorylation	3
General	Localization	415
	Binding	158
	Regulation	540
	Positive regulation	966
	Negative regulation	683
Planned	Planned process	582

## Conclusions

In this paper we develop a novel multi-source transfer learning approach for wide coverage event trigger recognition. We design a multiple channel structure based on adversarial networks to set the transfer learning, which can share knowledge between the source and target domains more comprehensively. Under the constraint of an extra classifier, the multiple channels can extract and transfer more diverse common features from the related multi-source domains effectively. In the experiments, our proposed transfer learning-based MSTLTR system achieves significant trigger recognition improvement. Moreover, performance is competitive compared with other leading trigger recognition systems using the same MLEE corpus. Hence this study contributes to effective recognition of biomedical trigger words from text across wide coverage. The effectiveness of this method is more prominent when the amount of data in the target area is scarce. For the future work, we plan to apply the multi-source transfer learning approach to more challenging biomedical text mining tasks such as event extraction, where multiple source datasets exist.

## Methods

In this section, we introduce our proposed multi-source transfer learning approach. Our solution for trigger recognition is mainly based on a Bidirectional LSTM-CRF model (BiLSTM-CRF) [39], which uses a Long Short Term Memory (LSTM) neural network [40] to extract features to train a Conditional Random Field (CRF) [41]. We embed a transfer learning approach to allow for joint training with multi-source datasets to improve the recognition performance effectively.

**Fig. 2:**
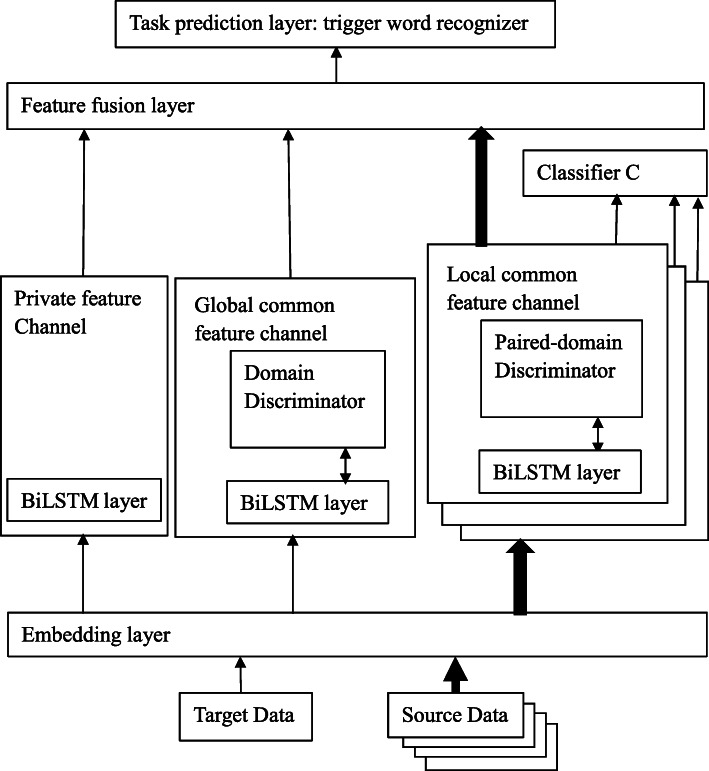
Our proposed Multi-Source Transfer Learning-based Trigger Recognizer (MSTLTR) framework

Our proposed transfer learning approach is inspired by the private-common feature method, where the features are divided into two parts: private features and common features. The private features are task specific ones extracted from target dataset. The common features are task invariant ones, which are extracted via adversarial networks trained on both the target and the source datasets. Through the common features, the useful knowledge can be transferred from one specific source domain to the target domain. In order to extend the transfer learning method of using one source data set to using multiple source datasets, and at the same time, try not to lose useful common feature information, we divide the common features into two parts: global and local common features. The global common features are extracted via an adversarial network trained on both the target and all the source datasets. Meanwhile, the local common features are extracted from paired adversarial networks based on pairs of the target and each source datasets. All the global common, local common and private features will pass through a fusion layer. There, the private features are augmented by the global common, local common features generated through transfer learning. The architecture of our model is shown in Fig. 2, which has a hierarchical structure.

### Model architecture

Figure 2 shows a sketch of our proposed Multi-Source Transfer Learning-based Trigger Recognizer (MSTLTR) framework. The model has six main modules: an embedding layer for word representation, a private feature channel based on a BiLSTM network, a global common feature channel based on an adversarial network, multiple local common feature channels based on paired adversarial networks and an extra classifier, a feature fusion layer, and a task prediction layer for trigger word recognition. For a given input sentence *s*={*word*_1_,*word*_2_,…*word*_*n*_}_*k*_ from domain *k*, the aim of trigger recognition is to output a tag sequence {*y*_1_,*y*_2_,…*y*_*n*_}_*k*_, where *word*_*i*_ is a word (or token) in the sentence and *y*_*i*_ denotes its corresponding type label. The value of *y*_*i*_ belongs to the label set, which is a biomedical event type or negative if it does not indicate any event. *k* is the domain number: when *k*=0, the input sentence comes from the target domain; when *k*=1,2,…,*K*, the input sentence comes from one of the source domains. All the main modules are explained below.

#### Embedding layer: word representation

In order to express both syntactic and semantic information in input sentences, word embedding, character embedding, part-of-speech (POS) embedding, named entity type embedding and dependency tree-based word embedding vectors are utilized to represent each word.

1. Word embedding vector *E*^*w*^: It maps each word in an input sentence to a word embedding vector *E*^*w*^, which contains semantic information from its linear contexts. We use pre-trained word embedding vectors learned from PubMed articles using the word2vec model [42].
2. Character embedding vector *E*^*c*^: We use an extra LSTM network to extract the orthographic information from the sequence of characters of each word. The LSTM network is initialized randomly and trained to output a character-level embedding vector *E*^*c*^.
3. POS embedding vector *E*^*p*^: We use the POS feature to extend the word representation. It maps the POS tag of each word in an input sentence to a POS embedding vector, which extracts syntactic information. We use the Gdep parsing tool [43] to extract POS tags for words in each sentence. Gdep is a dependency analysis tool for biomedical text, which can extract syntax annotation with high precision.
4. Named entity type embedding vector *E*^*e*^: It maps named entity type of each word in an input sentence to a embedding vector to extract domain-dependent information. The named entities are provided by the task data. In some cases, a certain named entity might span through multiple words. For the sake of simplicity of generating word embedding vector, we assign every word spanned by that named entity the same entity type.
5. Dependency tree-based word embedding vector *E*^*d*^: In order to extend features from linear contexts to non-linear syntactic contexts, each word from an input sentence is mapped to a dependency tree-based word embedding vector, which contains rich functional and syntactic information. In this paper, we use pre-trained dependent-based word embedding vectors learned from English Wikipedia using the skip-gram model [44].

#### Private feature channel: BiLSTM network

The private feature channel *Ch*_*PF*_ contains a BiLSTM network layer, which extracts private features from the specific domain of the input sentence. This layer takes a concatenation of the outputs of the previous embedding layer as input, $x_{i}=\left [E_{i}^{w};E_{i}^{c};E_{i}^{p};E_{i}^{e};E_{i}^{d}\right ]$. Because of the ability to learn long-distance dependencies in a sequence through memory cells, an LSTM is a powerful tool for sequence labeling tasks [40]. Suppose that an input to the LSTM layer is a sequence of embedding outputs {*x*_1_,*x*_2_,…,*x*_*T*_}. It produces an output sequence of {*h*_1_,*h*_2_,…,*h*_*T*_}. The following implementation strategy is employed during training [39], where both sequences have the same length *T*:

4 $$ i_{t}= \sigma\left(W_{{xi}}x_{t}+W_{{hi}}h_{t-1}+W_{{ci}}c_{t-1}+b_{i}\right)  $$

5 $$ f_{t}= \sigma(W_{{xf}}x_{t}+W_{{hf}}h_{t-1}+W_{{cf}}c_{t-1}+b_{f})  $$

6 $$ c_{t}=f_{t}c_{t-1} + i_{t}tanh(W_{{xc}}x_{t} + W_{{hc}}h_{l-1} + b_{c})  $$

7 $$ o_{t}= \sigma(W_{{xo}}x_{t} + W_{{ho}}h_{t-1} + W_{{co}}c_{t} + b_{o})  $$

8 $$ h_{t}= o_{t}tanh(c_{t})  $$

where *σ* and *tanh* denote the logistic sigmoid function and the hyperbolic tangent activation function, respectively. All *W*s and *b*s are weights and biases of LSTM, which are the trainable parameters (*θ*_*p*_) of *Ch*_*PF*_. More details about memory cells can be referred to in [39]. In sequence labelling tasks, it’s better to be able to process both past and future context dependencies in the sequence. Therefore, Bidirectional LSTM (BiLSTM) [39, 45], another version of LSTM, is commonly employed. In BiLSTM, the forward LSTM captures features from the left side (past) and the backward LSTM captures features from the right side (future) for each word. So, each word effectively encodes information about the whole sentence. The output of the private feature channel, private features *F*_*p*_, is obtained by concatenating the outputs of the forward and backward LSTMs $F_{p}=\left [h_{t}^{F};h_{t}^{B}\right ]$.

#### Global common feature channel: adversarial network

The global common feature channel *Ch*_*GF*_ is built on an adversarial network [21]. The adversarial network contains components of a feature extractor and a domain discriminator *DC*. The feature extractor is the same BiLSTM network used in the private feature channel. And it produces global common features, $F_{g}=\left [h_{t}^{F};h_{t}^{B}\right ]$. *DC* is a domain classifier that takes the global common features of an input sentence and trained to identify which dataset the input sentence belongs to. Formally, the *DC* function can be expressed as follows:

9 $$ DC\left(F_{g}\right) = softmax\left(W_{{DC}}F_{g}+b_{{DC}}\right)  $$

where *W*_*DC*_ and *b*_*DC*_ are weights and biases, which can be denoted as trainable parameters *θ*_*DC*_. *F*_*g*_, the global common features extracted through *Ch*_*GF*_, is the input of the *DC* function.

We employ a multi-class version of adversarial network in *Ch*_*GF*_ [24]. The adversarial loss in *Ch*_*GF*_ is defined as *L*_*gAdv*_. It trains the *Ch*_*GF*_ to prevent domain specific features into the global common features among multiple domains. The objective of *Ch*_*GF*_ that entails the optimization of *L*_*gAdv*_ can be expressed as follows:

10 $$ {}J_{{gAdv}} = \min_{\theta_{g}}\max_{\theta_{{DC}}} L_{{gAdv}} = \mathbb{E}\left[\sum\limits_{k=0}^{K} \sum\limits_{i=1}^{T_{k}}d_{k}^{i} \log\left[D\left(F_{g}\left(x_{k}^{i}\right)\right)\right]\right]  $$

where *θ*_*g*_ denotes the trainable parameters of BiLSTM of *Ch*_*GF*_. *T*_*k*_ is the number of training instances of the domain *k*, and $x_{k}^{i}$ is the *i*^*th*^ sentence from domain *k*. $d_{k}^{i}$ denotes the corresponding ground-truth label indicating the domain of the current input. In the training phase, there is minimax optimization, and the *DC* is driven to reach a point where the domains cannot be differentiated based on the extracted *F*_*g*_. After training, since *DC* cannot identify the domain of the input sentence, the *F*_*g*_ then do not carry domain related information among the target and all the source datasets. Hence, the *F*_*g*_ is domain-invariant and output through *Ch*_*GF*_.

#### Local common feature channel: paired adversarial networks and extra classifier

The local common feature channel (*Ch*_*LF*_) is designed with an architecture consisting of a set of paired adversarial networks and an extra classifier. In addition to the private features obtained from the target domain, the common features are obtained through transfer learning in *Ch*_*GF*_. However, when there is more than one source domain from different recognition tasks available, we should be able to get more common features than *Ch*_*GF*_ provides. Some useful sharable features could be ignored by *Ch*_*GF*_. Therefore, *Ch*_*LF*_ is proposed using multi-source transfer learning to address the problem to provide a more comprehensive shared feature representation. The hypothesis is the case that the more abundant features are provided from the source domains through transfer learning, the better the recognition performance will be in the target domain. *Ch*_*LF*_ has multiple sub-channels, and each sub-channel $Ch_{LF_{j}}$ has a similar adversarial network to *Ch*_*GF*_.

The key difference is that each sub-channel $Ch_{LF_{j}}$ is a paired adversarial network. Its *DC*_*j*_ component is built based on the domain pair composed of the target domain and one of the source domains *j*. Hence, one local common feature sub-channel only extracts the common features between the target domain and a certain source domain. The local common feature vector is denoted as $F_{l_{j}}$. The paired-adversarial loss in each $Ch_{LF_{j}}$ is defined as $L_{pair-Adv_{j}}$ to prevent domain specific features into the local common features from this channel. Hence, the $L_{pair-Adv_{j}}$ trains each $Ch_{LF_{j}}$ to produce the local common features such that each *DC*_*j*_ cannot reliably recognize which domain the sentence comes from. The objective that entails the optimization of the loss $L_{pair-Adv_{j}}$ can be expressed as follows:

11 $$ {}\begin{aligned} J_{pair-Adv_{j}} &= \min_{\theta_{l_{j}}}\max_{\theta_{DC_{j}}} L_{pair-Adv_{j}}\left(S_{j},T\right)\\ &= \mathbb{E}\left[ \sum\limits_{k=1}^{2} \sum\limits_{i=1}^{T_{k}}d_{k}^{i} \log\left[D\left(F_{l_{j}}\left(x_{k}^{i}\right)\right)\right]\right] \end{aligned}  $$

where $\theta _{l_{j}}$ and $\theta _{DC_{j}}$ denote the trainable parameters of BiLSTM and discriminator of each $Ch_{LF_{j}}$. In the paired domain discrimination mode, the number of domains is 2, the target domain and the source domain *j*. After training, each $F_{l_{j}}$ then is output through $Ch_{LF_{j}}$, respectively.

Moreover, to prevent local common features from interfering with each other, an extra classifier *C* is designed to identify from which source domain the generated $F_{l_{j}}$ comes. The classifier *C* induces constraint. The objective of *C* entails the minimization of the loss can be expressed as follows:

12 $$ J_{C} = \min_{\theta_{C}} = \mathbb{E}\left[\sum\limits_{j=1}^{K} \sum\limits_{i=1}^{T_{j}}d_{j}^{i}\log\left[C\left(F_{l_{j}}\left(x_{j}^{i}\right)\right)\right]\right]  $$

where *θ*_*C*_ denotes the trainable parameters of classifier *C*, and $d_{j}^{i}$ denotes the corresponding ground-truth label indicating the channel of the current input.

Then the total objective of the local common feature channel, *J*_*lAdv*_, is defined as follows:

13 $$ J_{{lAdv}} = \frac{1}{K} \sum\limits_{j=1}^{K}J_{pair-Adv_{j}} - \beta J_{C}  $$

where *K* is the number of $Ch_{LF_{j}}$s, and at the same time it also is the number of source domains. And hyper-parameter *β* controls the effects of *C* on feature extraction.

#### Feature fusion layer

The features used to predict trigger word labels are provided through multiple feature channels, including one private feature channel, one global common feature channel and several local common feature sub-channels. Before label prediction, all these features will be fused properly in this layer. The concatenation of the output of each feature channel, $F_{{con}}=\left [F_{p};F_{g};F_{l_{1}};F_{l_{2}};\ldots ;F_{l_{K}}\right ]$, is mapped through a linear and fully-connected network layer to obtain the final feature vector *F* through a hyperbolic tangent activation function.

14 $$ F = \tanh\left(W_{{FF}}F_{{con}} + b_{{FF}}\right)  $$

where *W*_*FF*_ and *b*_*FF*_ are trainable parameters of feature fusion layer, denoted as *θ*_*FF*_.

#### Task prediction layer: trigger word recognizer

On the top of the feature fusion layer, a final trigger recognizer is built through a CRF layer generating a sequence of labels for corresponding words. The CRF layer can learn the strong dependencies across output labels and come into the most likely sequence of predicted tags [46]. Whenever given a feature vector *F* of the input sentence with a label sequence *y*=(*y*_1_,*y*_2_,…,*y*_*n*_), the objective of the recognition task (RT) loss function *L*_*RT*_ to be maximized can be defined as follows:

15 $$ J_{{RT}} = \max_{\theta_{{crf}}} \left[f\left(F,y\right)- \log \sum exp\left(f\left(F,\hat{y}\right)\right)\right]  $$

where *θ*_*crf*_ is the trainable parameters of CRF layer, $\hat {y}$ is the predicted label sequence according to *y*, and *f* is the defined score function.

### Model training

The overall objective function of our model can be computed as the follows:

16 $$ J = J_{{RT}} + \alpha_{1} J_{{gAdv}} + \alpha_{2} J_{{lAdv}}  $$

where *α*_1_ and *α*_2_ are hyper-parameters to control the transferring of the global and local common features.

In the training phase, at each iteration, we first select a batch of training instances from the target or one of the source domain in turn, which is used to update the parameters of the model. For each batch of training instances, there are three optimizers need training. The first one is to calculate *J*_*RT*_ with the parameters of *θ*_*p*_,*θ*_*FF*_ and *θ*_*crf*_. The second one is to calculate *J*_*gAdv*_ with the parameters of *θ*_*g*_ and *θ*_*DC*_. The third one is to calculate *J*_*lAdv*_ with the parameters of $\theta _{l_{j}}, \theta _{DC_{j}}$, and *θ*_*C*_. Finally, *J* are optimized through above three steps, and all the parameters are updated with backpropagation. We repeat the above optimizing iterations until convergence of the target domain.

### Basic model

**Fig. 3:**
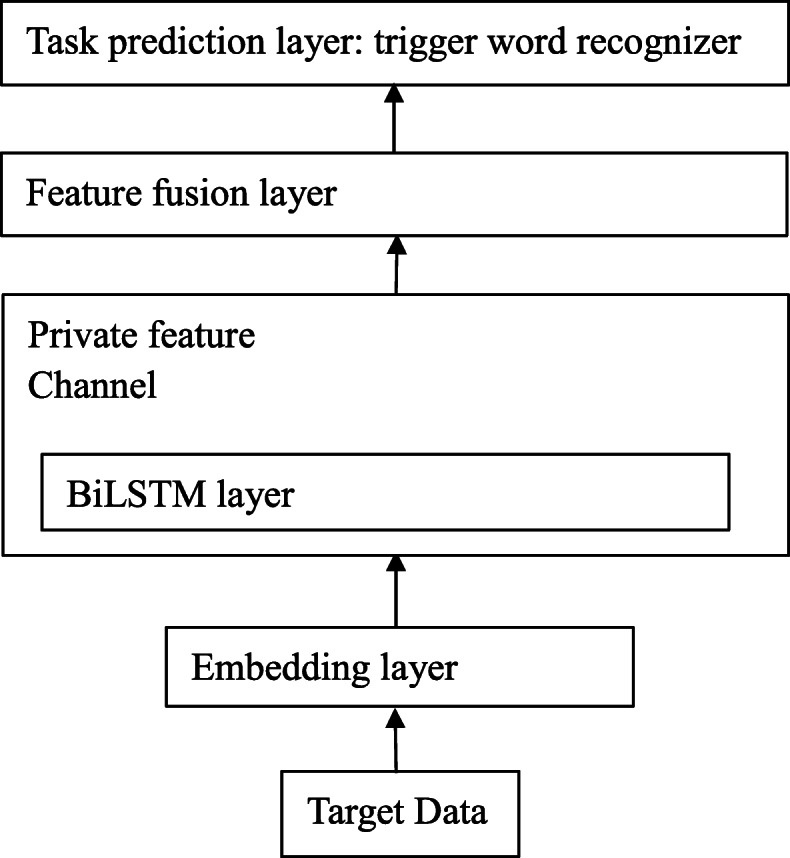
The Basic Model framework

If we remove the transfer learning modules of the global and local common feature channels from the MSTLTR model, we will get a Basic Model. The Basic Model doesn’t use any transferred common features, which provides a baseline performance for comparison. The architecture of the Basic Model is shown in Fig. 3. After the word embedding layer, because there are no common features provided by the global and local common feature channels from source domain data, only the private feature channel is valid. In the feature fusion layer, a fully connected network only receives these private features and transmits them to the prediction layer for trigger word recognition. Therefore, removing the optimization of *J*_*gAdv*_ and *J*_*lAdv*_, the overall objective function of the Basic Model can be computed as the follows:

17 $$ { J = J_{{RT}}}  $$

In the training phase, only *J*_*RT*_ with the parameters of *θ*_*p*_,*θ*_*FF*_ and *θ*_*crf*_ need to be optimized until convergence on the target domain.

## Data Availability

The MLEE corpus analysed during the current study is available in http://www.nactem.ac.uk/MLEE/#availability[8]. The corpus from the BioNLP’09 Shared Tasks is available in http://www.geniaproject.org/shared-tasks/bionlp-shared-task-2009[4]. The corpus from the Epigenetics and Post-translational Modifications (EPI) task of BioNLP challenge 2011 is available in http://weaver.nlplab.org/~bionlp-st/BioNLP-ST/downloads/downloads.shtml[5]. The corpus from the Infectious Diseases (ID) task of BioNLP challenge 2011 is available in http://weaver.nlplab.org/~bionlp-st/BioNLP-ST/downloads/downloads.shtml[5]. The corpus about DNA methylation and demethylation events recognition is available in http://www.geniaproject.org/other-corpora/dna-methylation-corpus[6].

## Abbreviations

TM: Text mining
CRF: Conditional random field
SVM: Support vector machine
ANN: Artificial neural network
NLP: Natural language processing
TL: Transfer learning
MSTLTR: Multi-source transfer learning-based trigger recognizer
ST: Shared task
EPI: Epigenetics and post-translational modifications
ID: Infectious diseases
RT: Recognition task
NER: Named entity recognition
POS: Part-Of-speech
LSTM: Long short term memory
BiLSTM: Bidirectional LSTM
BiLSTM-CRF: Bidirectional LSTM-CRF model
CNN: Convolution neural network
RNN: Recurrent neural network

## Footnotes

1 ^*1*^https://github.com/dmis-lab/biobert
